# Supplementary material for: Apolipoprotein E controls Dectin-1-dependent development of monocyte-derived alveolar macrophages upon pulmonary β-glucan-induced inflammatory adaptation
Source: Nat Immunol. 2024 Apr 26;25(6):994–1006. doi: 10.1038/s41590-024-01830-z (PMC11147775; doi:10.1038/s41590-024-01830-z)
Supplement: Supplementary file 1 — Supplementary Figs. 1–7. [file 41590_2024_1830_MOESM1_ESM.pdf]

nature immunology

Article

https://doi.org/10.1038/s41590-024-01830-z

Apolipoprotein E controls Dectin-1-dependent development of monocyte-derived alveolar macrophages upon pulmonary β-glucan-induced inflammatory adaptation

In the format provided by the authors and unedited

Supplement Figure 1

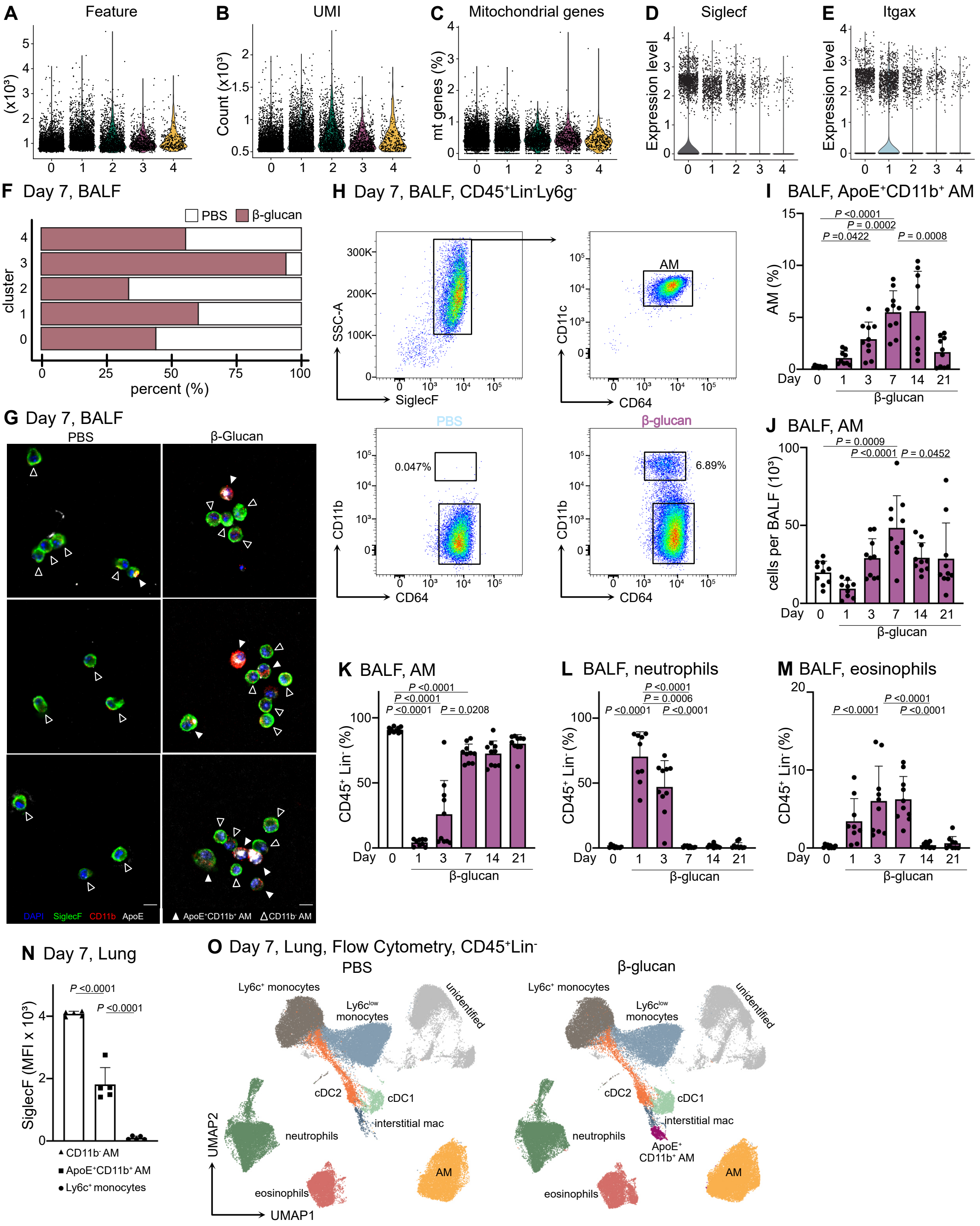

Supplement Figure 2

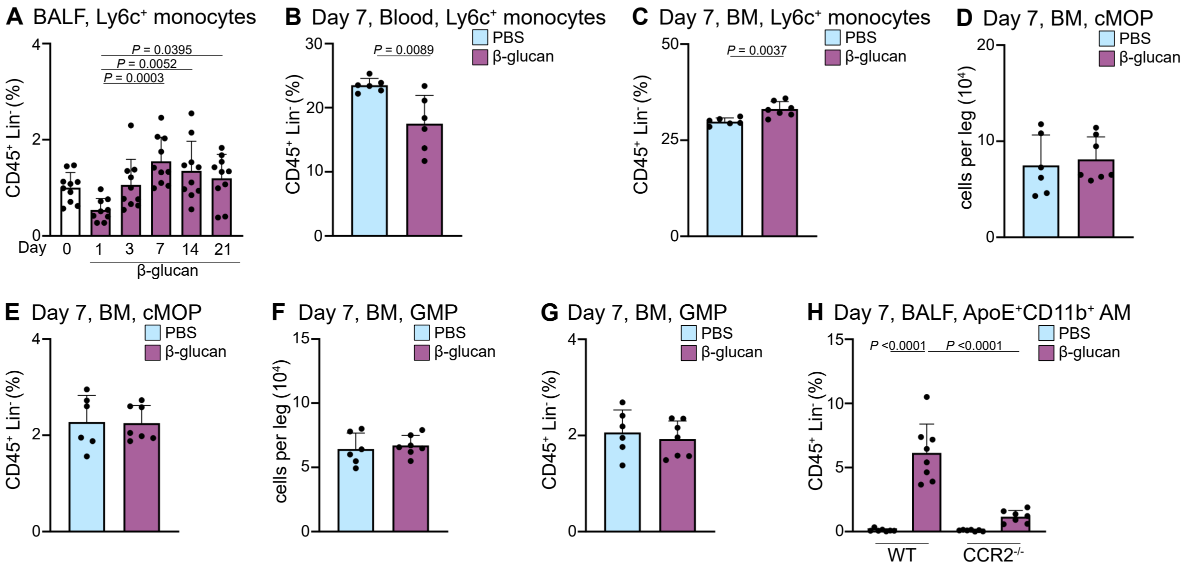

Supplement Figure 3

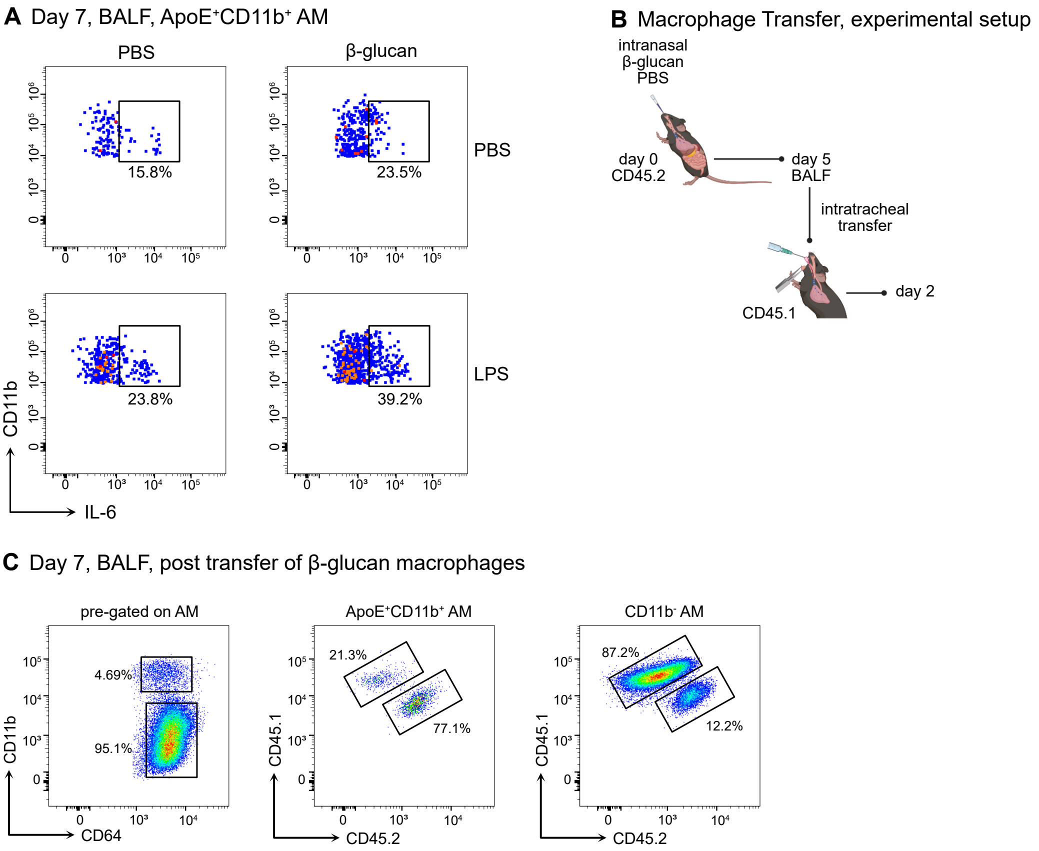

Supplement Figure 4

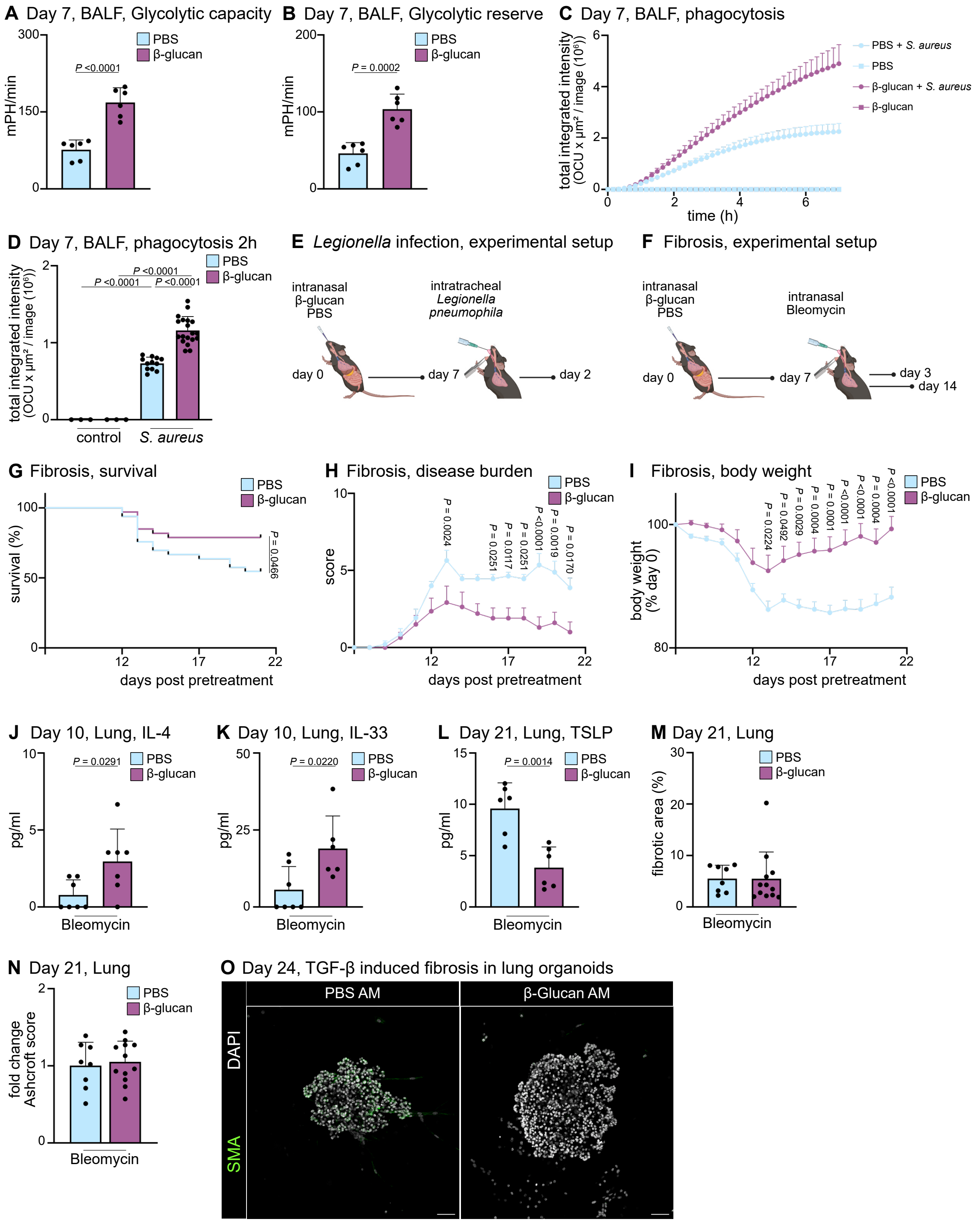

Supplement Figure 5

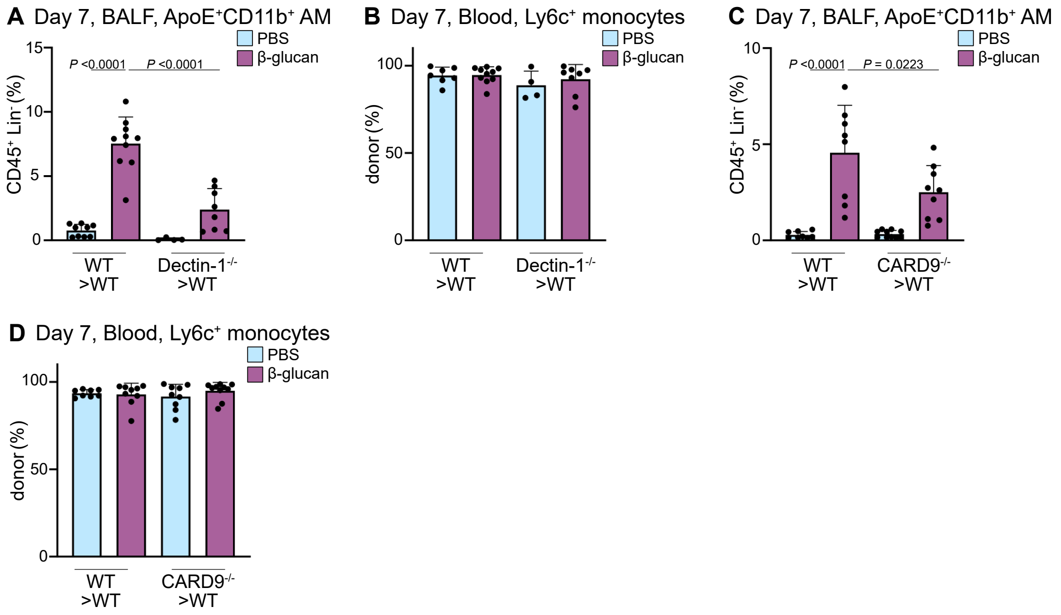

Supplement Figure 6

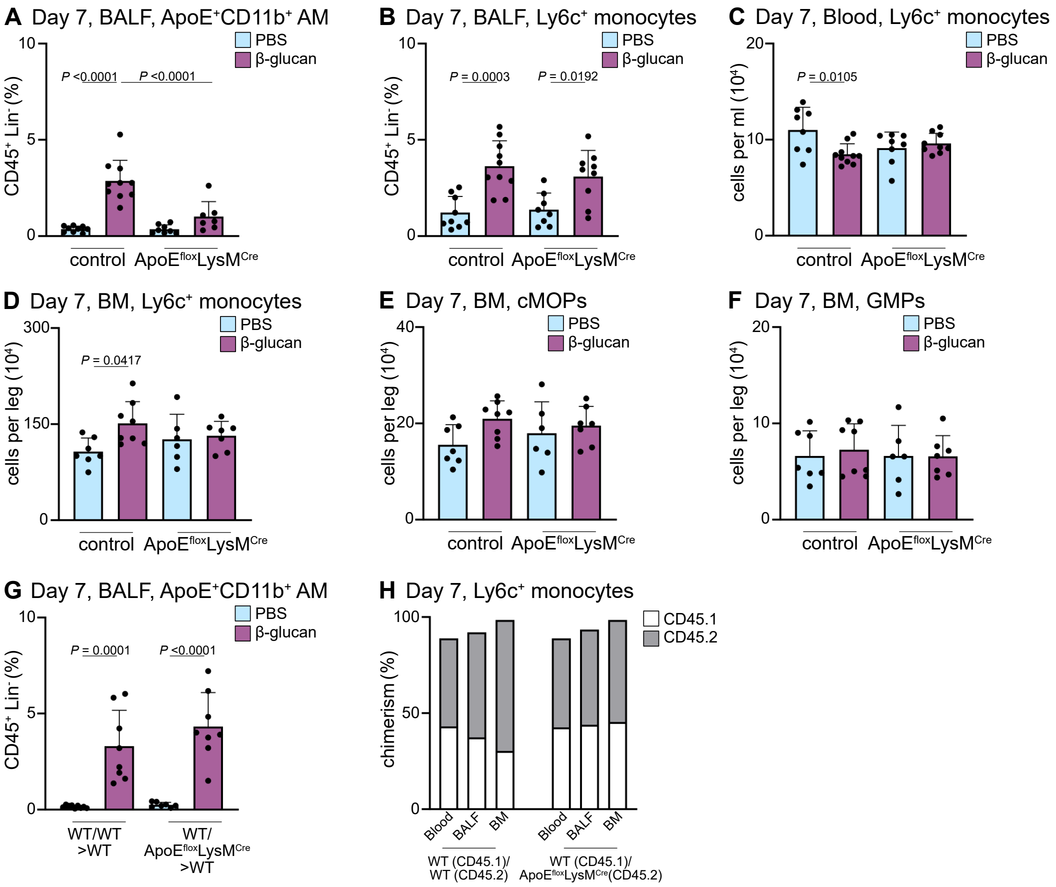

Supplement Figure 7

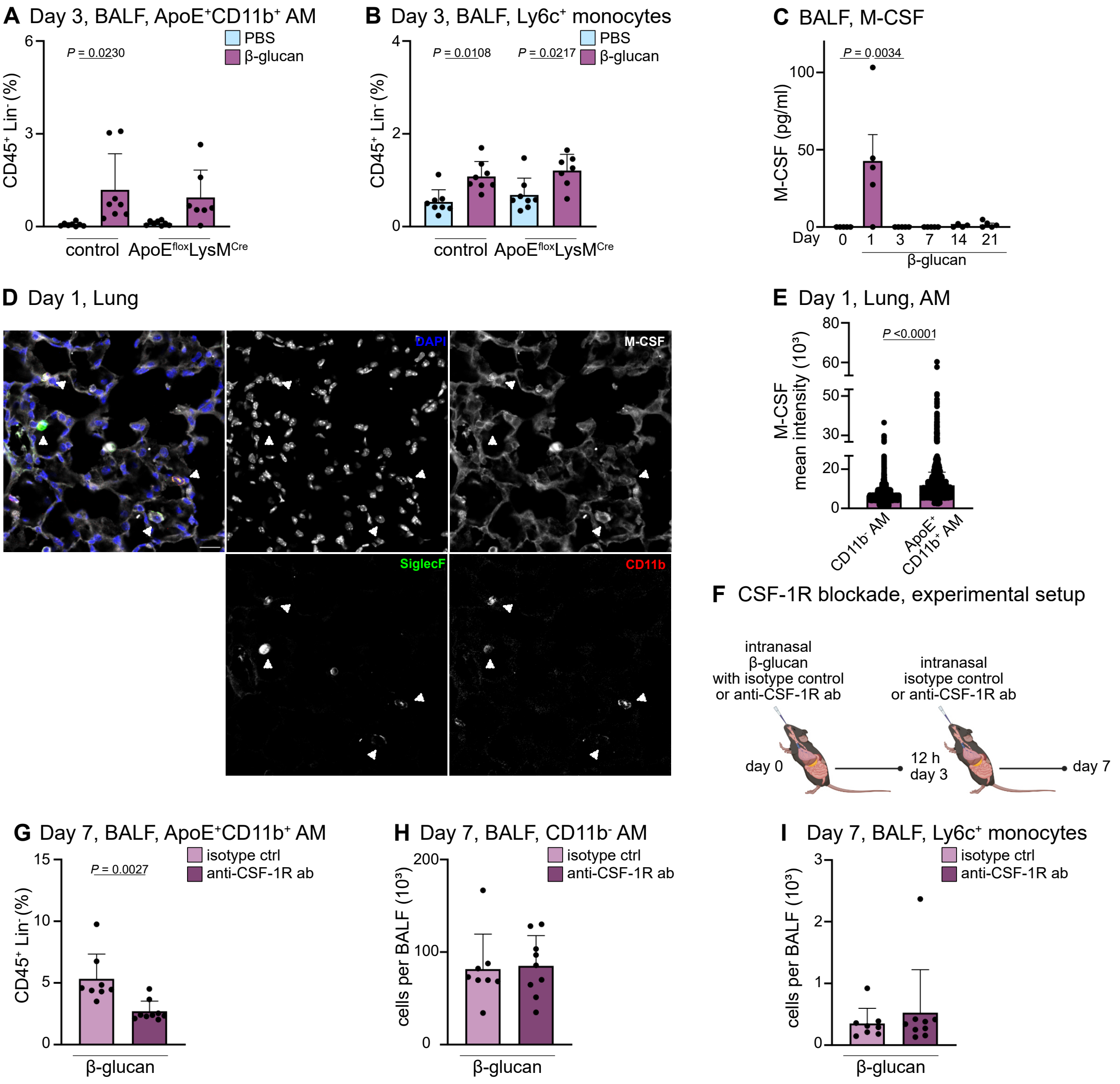

Supplemental Figure Legends

Supplemental Figure 1 to Figure 1

(A-F) ScRNA sequencing of the BALF seven days after intranasal stimulation with PBS or β-glucan in wt mice. (A) Violin plot of feature gene counts per cluster. (B) Violin plot of UMI counts per cluster. (C) Violin plot of mitochondrial gene percentage per cluster. (D, E) Violin plot of *Siglecf* (D) and *Itgax* (E) RNA expression levels. (F) Percentage of contribution of condition, PBS or β-glucan, to the five clusters. (G) AM of PBS or β-glucan treated WT mice were selected by adherence from the BALF 7 days after exposure and subsequently stained with a 17-plex CODEX antibody panel. Overlay image shows the markers used to identify AM populations. Filled arrows indicate $ApoE^+CD11b^+$ AMs, whereas open arrows $CD11b^-$ AMs. Scale bar represents 10 μm (n= 2 mice pooled, two independent experiments). (H) Flow cytometry gating strategy pre-gated on Lin^- (Ly6g, B220, CD19, CD3ε, Nk1.1, Ter-119) and $CD45^+$ cells to define $ApoE^+CD11b^+$ AM in the BALF after intranasal PBS or β-glucan stimulation. (I) Flow cytometric quantification of $ApoE^+CD11b^+$ AM numbers as frequency among AM in the BALF in a time course from 1 to 21 days post β-glucan stimulation of wt mice (n= 9-10 mice, two independent experiments). (J, K) Flow cytometric quantification of absolute numbers (J) or frequency (K) of AM ($CD45^+SiglecF^+CD64^+CD11c^+$) among $CD45^+Lin^-$ cells in the BALF in a time course from 1 to 21 days post β-glucan stimulation of wt mice (n= 9-10 mice, two independent experiments). (L, M) Flow cytometric quantification of frequency of neutrophils (L) and eosinophils (M) among $CD45^+Lin^-$ cells in the BALF in a time course from 1 to 21 days post β-glucan stimulation of wt mice (n= 9-10 mice, two independent experiments). (N) Mean fluorescence Intensity (MFI) of SiglecF on indicated cell subsets in lung seven days post stimulation with β-glucan (n=4 mice, two independent experiments). (O) UMAP representation of intranasally stimulated lungs with PBS or β-glucan seven days after stimulation (pooled n=3 mice per condition). Data are depicted as mean ± SD, (I-N) ordinary one-way ANOVA with Tukey's multiple comparisons.

Supplemental Figure 2 to Figure 2

(A) Quantification of frequency of $Ly6c^+$ monocytes among $CD45^+Lin^-$ cells in the BALF in a time course from 1 to 21 days post β-glucan stimulation of WT mice by flow cytometry (n= 9-10 mice, two independent experiments). (B, C) Percentage of $Ly6c^+$ monocytes among $CD45^+Lin^-$ cells in the blood (B) or in the BM (C) of WT mice seven days after PBS or β-glucan by flow cytometry (n = 6 mice, two individual experiments). (D-G) Frequency among $CD45^+Lin^-$ cells and absolute numbers of cMOP (D, E) and GMP (F, G) in the BM of WT mice seven days after PBS or β-glucan by flow cytometry (n = 6 mice, two individual experiments). (H)

Quantification of $ApoE^+CD11b^+$ AM frequency among $CD45^+Lin^-$ cells in the BALF seven days after β-glucan exposure in WT or $CCR2^{-/-}$ mice (n=7-8 mice, two individual experiments) by flow cytometry. Data are depicted as mean ± SD, (A, H) ordinary one-way ANOVA with Tukey's multiple comparisons, (B-G) unpaired two-tailed student's *t*-test.

Supplemental Figure 3 to Figure 3

(A) BALF cells were harvested from WT mice seven days after intranasal exposure with PBS or β-glucan and subsequently restimulated *in vitro* with 10 ng/ml LPS. Representative flow cytometry dot plots of $IL\text{-}6^+$ $ApoE^+CD11b^+$ AM after restimulation with LPS for 6-8 h followed by intracellular staining gated on $ApoE^+CD11b^+$ AM (n=6 mice, two independent experiments). (B) Experimental setup of macrophage transfer. PBS or β-glucan experienced $CD45.2^+$ WT macrophages were harvested five days after stimulation and intratracheally transferred into $CD45.1^+$ mice. Readout per flow cytometry or *in vitro* restimulation 48 h after transfer. (C) Representative flow cytometry plot of the BALF of $CD45.1^+$ recipient mice 48 h post transfer of β-glucan experienced $CD45.2^+$ donor macrophages.

Supplemental Figure 4 to Figure 4

(A, B) Measurement of glycolytic reserve (A) and glycolytic capacity (B) in BALF cells seven days after PBS or β-glucan stimulation by extracellular flux analysis (n=6 mice, one independent experiment). (C, D) AM cells from day seven PBS or β-glucan experienced WT mice were selected by adherence and subsequently treated with 2.5 µm pHrodo *S.aureus* bioparticles (n=3 mice pooled per condition, technical replicates: 6 control wells, 12-19 treated wells per group, one out of two independent experiments shown). (C) Total integrated intensity (OCU x μm^2/image) of the phagocytosis signal in AM over the time course of 7 h (here shown as mean ±SD of all technical replicates). (D) Quantification of the total integrated intensity in $phagocytosis^+$ AM 2 h after adding the pHrodo *S.aureus* bioparticles. (E) Experimental setup of an acute secondary bacterial infection. Intranasal PBS or β-glucan stimulation at day zero was followed by intratracheal inoculation with *Legionella pneumophila* at day seven and sacrifice two days after infection. (F) Experimental setup of a chronic secondary fibrosis challenge. Intranasal PBS or β-glucan stimulation at day 0 was followed by intratracheal application of *Streptomyces verticillus* bleomycin at day seven and sacrifice three or 14 days after fibrosis induction. (G-I) Health assessment of mice after pretreatment with PBS or β-glucan (day 0) prior to fibrosis induction (day 7). (G) Kaplan-Meier plot of survival (n=35 mice, two independent experiments). (H) Disease burden (general health appearance, spontaneous

behavior, body weight) (n=14 mice, one representative experiment from two independent experiments shown). (I) Body weight as % change based on the initial weight at day 0 (n=14 mice, one representative experiment from two independent experiments shown). (J-L) Quantification of IL-4 (J), IL-33 (K) and TSLP (L) protein levels 3 or 14 days after bleomycin treatment (n=6-7 mice, one independent experiment). (M, N) Quantification of the fibrotic area as percent of the total lung tissue area (M) and the fold change of Ashcroft score (N) by histology 14 days after bleomycin treatment (n=8-11 mice, two independent experiments). (O) Representative confocal images of lung BALOs co-cultured with PBS- or β-glucan-experienced AM. As a control to TGF-β, AM-BALO co-cultures were treated with medium only. After fixation, co-cultures were stained for α-SMA (n=6-8 organoids per condition from two replicate wells, one out of two independent experiments shown). Scale bars represent 50 µm. Data are depicted as mean ± SD, (A-B, L-N) unpaired two-tailed student's *t*-test, (D) ordinary one-way ANOVA with Tukey's multiple comparisons, (G) Mantel-Cox test, (H, I) ordinary two-way ANOVA with Šídák's multiple comparisons.

Supplemental Figure 5 to Figure 5

(A, B) Frequency of $ApoE^{+}CD11b^{+}$ AM among $CD45^{+}Lin^{-}$ cells in the BALF (A) seven days after PBS or β-glucan exposure in $Dectin1^{-/-}$ BM chimeras and the respective blood chimerism for $Ly6c^{+}$ monocytes (B) assessed by flow cytometry (n=4-10 mice, two independent experiments). (C, D) Frequency of $ApoE^{+}CD11b^{+}$ AM among $CD45^{+}Lin^{-}$ cells in the BALF (C) seven days after PBS or β-glucan exposure in $CARD9^{-/-}$ BM chimeras and the respective blood chimerism for $Ly6c^{+}$ monocytes (D) assessed by flow cytometry (n=8-9 mice, two independent experiments). Data are depicted as mean ± SD, (A, C) ordinary one-way ANOVA with Tukey's multiple comparisons.

Supplemental Figure 6 to Figure 6

(A, B) Frequency of $ApoE^{+}CD11b^{+}$ AM (A) and $Ly6c^{+}$ monocytes (B) among $CD45^{+}Lin^{-}$ cells seven days after intranasal β-glucan exposure of control or $ApoE^{flox}LysM^{Cre}$ mice by flow cytometry (n=8-10 mice, three independent experiments). (C-F) Absolute numbers of $Ly6c^{+}$ monocytes in the blood (C) or BM (D), cMOP (E) and GMP (F) in the BM of control or $ApoE^{flox}LysM^{Cre}$ mice seven days after intranasal β-glucan exposure quantified by flow cytometry (n=6-8 mice, two independent experiments). (G, H) Lethally irradiated $CD45.1^{+}/CD45.2^{+}$ male mice were reconstituted with $1.5x10^{6}$ $CD45.1^{+}$ mixed with $CD45.2^{+}$ BM cells (WT/WT) or with $CD45.1^{+}$ mixed with $ApoE^{flox}LysM^{Cre}$ $CD45.2^{+}$ BM cells (WT/ $ApoE^{flox}LysM^{Cre}$) for 12 weeks and

subsequently intranasally stimulated with PBS or β-glucan (n=8-9 mice, two independent experiments). Flow cytometric quantification of ApoE$^+$CD11b$^+$ AM frequency (G) among CD45$^+$Lin$^-$ cells and chimerism of Ly6c$^+$ monocytes in blood, BALF and BM (H). Bars in (H) are further subdivided into CD45.1$^+$ or CD45.2$^+$ to display contribution of transferred mixed BM donors. Data are depicted as mean ± SD, (A-G) ordinary one-way ANOVA with Tukey's multiple comparisons.

Supplemental Figure 7 to Figure 7

(A) Frequency of ApoE$^+$CD11b$^+$ AM (A) and Ly6c$^+$ monocytes (B) among CD45$^+$Lin$^-$ cells three days after intranasal β-glucan exposure in control or ApoE^{flox}LysM^{Cre} mice (n=7-8, two independent experiments) by flow cytometry. (C) Quantification of M-CSF protein levels in the BALF in a time course from one to 21 days post β-glucan stimulation of WT mice (n=5 mice, one independent experiment). (D, E) Lungs from WT mice were harvested 24 h after β-glucan stimulation, fixed, and frozen in OCT. 5 μm sections were prepared and stained with antibodies to identify AMs and visualize M-CSF production. Representative images are shown in (D). Arrows indicate ApoE$^+$CD11b$^+$ AMs. Seven regions of the same size from samples in (D) were acquired and analyzed as explained in Materials and Methods. Plot in (E) shows the M-CSF mean signal intensities of CD11b$^-$ and ApoE$^+$CD11b$^+$ AMs. Each dot represents an individual cell. (F) Experimental setup of *in vivo* CSF-1R blockade. WT mice were intranasally stimulated with β-glucan mixed with an anti-CSF-1R antibody or the respective isotype control and repeatedly treated 12 h and 3 days after initial stimulation. (G- I) Frequency of ApoE$^+$CD11b$^+$ AM (G) among CD45$^+$Lin$^-$ cells and absolute numbers of AM (H) and Ly6c$^+$ high monocytes (I) in the BALF of WT mice seven days after intranasal β-glucan treatment together with 500 μg of CSF-1R antibody or the respective isotype control (n=8-9 mice, two independent experiments). Data are depicted as mean ± SD, (A-C) ordinary one-way ANOVA with Tukey's multiple comparisons, (E) Two-tailed Mann-Whitney test, (G-I) unpaired two-tailed student's *t*-test.
