# Supplementary material for: Apolipoprotein E controls Dectin-1-dependent development of monocyte-derived alveolar macrophages upon pulmonary β-glucan-induced inflammatory adaptation
Source: Nat Immunol. 2024 Apr 26;25(6):994–1006. doi: 10.1038/s41590-024-01830-z (PMC11147775; doi:10.1038/s41590-024-01830-z)
Supplement: Supplementary file 2 — Reporting Summary [file 41590_2024_1830_MOESM2_ESM.pdf]

nature portfolio

Corresponding author(s): Andreas Schlitzer

Last updated by author(s): Mar 13, 2024

Reporting Summary

Nature Portfolio wishes to improve the reproducibility of the work that we publish. This form provides structure for consistency and transparency in reporting. For further information on Nature Portfolio policies, see our Editorial Policies and the Editorial Policy Checklist.

Statistics

For all statistical analyses, confirm that the following items are present in the figure legend, table legend, main text, or Methods section.

n/a	Confirmed	
☐	☒	The exact sample size (*n*) for each experimental group/condition, given as a discrete number and unit of measurement
☐	☒	A statement on whether measurements were taken from distinct samples or whether the same sample was measured repeatedly
☐	☒	The statistical test(s) used AND whether they are one- or two-sided<br>*Only common tests should be described solely by name; describe more complex techniques in the Methods section.*
☐	☒	A description of all covariates tested
☐	☒	A description of any assumptions or corrections, such as tests of normality and adjustment for multiple comparisons
☐	☒	A full description of the statistical parameters including central tendency (e.g. means) or other basic estimates (e.g. regression coefficient) AND variation (e.g. standard deviation) or associated estimates of uncertainty (e.g. confidence intervals)
☐	☒	For null hypothesis testing, the test statistic (e.g. *F*, *t*, *r*) with confidence intervals, effect sizes, degrees of freedom and *P* value noted<br>*Give P values as exact values whenever suitable.*
☒	☐	For Bayesian analysis, information on the choice of priors and Markov chain Monte Carlo settings
☒	☐	For hierarchical and complex designs, identification of the appropriate level for tests and full reporting of outcomes
☒	☐	Estimates of effect sizes (e.g. Cohen's *d*, Pearson's *r*), indicating how they were calculated

Our web collection on statistics for biologists contains articles on many of the points above.

Software and code

Policy information about availability of computer code

Data collection	BD Diva, Sartorius Incucyte 2022A, BD Aria III, Tecan Infinite M200, Zeiss Axio Observer, Zeiss LSM 880 Airyscan, Akoya CODEX, Leica Stellaris 8, Illumina NextSeq500, Agilent Wave v2.1, TapeStation Analysis vA.02.01
Data analysis	FlowJo v10.8.1, GraphPad Prism v10, Microsoft Excel v2016, R Studio v1.1.463, R v4.1.3., Tecan i-control v1.12, CODEX MAV, CODEX Processor v1.7.0.6, CellProfiler, QuPath v0.3, bcl2fastq2 v2.20, Drop-seq v2.0.0, Seurat v4.1.1, ImageJ v2

For manuscripts utilizing custom algorithms or software that are central to the research but not yet described in published literature, software must be made available to editors and reviewers. We strongly encourage code deposition in a community repository (e.g. GitHub). See the Nature Portfolio guidelines for submitting code & software for further information.

Data

Policy information about availability of data

All manuscripts must include a data availability statement. This statement should provide the following information, where applicable:

- Accession codes, unique identifiers, or web links for publicly available datasets
- A description of any restrictions on data availability
- For clinical datasets or third party data, please ensure that the statement adheres to our policy

Single-cell RNA sequencing reads were mapped to mouse reference genome mm10. Raw data can be accessed with GEO number "GSE211575". Scripts to generate all results and figures can be found at: https://github.com/schlitzerlab/trained_immunity_2022

Research involving human participants, their data, or biological material

Policy information about studies with human participants or human data. See also policy information about sex, gender (identity/presentation), and sexual orientation and race, ethnicity and racism.

Reporting on sex and gender	not applicable
Reporting on race, ethnicity, or other socially relevant groupings	not applicable
Population characteristics	not applicable
Recruitment	not applicable
Ethics oversight	not applicable

Note that full information on the approval of the study protocol must also be provided in the manuscript.

Field-specific reporting

Please select the one below that is the best fit for your research. If you are not sure, read the appropriate sections before making your selection.

☒ Life sciences ☐ Behavioural & social sciences ☐ Ecological, evolutionary & environmental sciences

For a reference copy of the document with all sections, see nature.com/documents/nr-reporting-summary-flat.pdf

Life sciences study design

All studies must disclose on these points even when the disclosure is negative.

Sample size	No statistical method was used to predetermine sample size. Each flow cytometry experiment was performed with at least 3-5 mice per group per replicate, which is commonly accepted in the field of immunology.
Data exclusions	Animal experiments: For bone marrow chimeras, mice were excluded from analysis if chimerism was <85%.<br>Flow cytometry: no data was excluded.<br>Single cell RNA sequencing: Cells that did not pass quality control (>10% mitochondrial genes, <500 or >3000 genes per cell) were excluded.
Replication	All experiments were successfully replicated and gave similar results. The number of replicates is indicated in the figure legends.
Randomization	Mice were randomly assigned to groups by genotype and treatment prior to the initiation of the experiment.
Blinding	Sample preparation, data acquisition and analysis was done blinded using the mouse ID. For histological scoring of fibrosis, sections were analyzed by two independent investigators that were blinded to the treatment. Single cell sequencing data analysis was performed by a bioinformatician, who had limited knowledge of the conditions prior to analysis.

Reporting for specific materials, systems and methods

We require information from authors about some types of materials, experimental systems and methods used in many studies. Here, indicate whether each material, system or method listed is relevant to your study. If you are not sure if a list item applies to your research, read the appropriate section before selecting a response.

Materials & experimental systems

n/a	Involved in the study	
☐	☒	Antibodies
☒	☐	Eukaryotic cell lines
☒	☐	Palaeontology and archaeology
☐	☒	Animals and other organisms
☒	☐	Clinical data
☒	☐	Dual use research of concern
☒	☐	Plants

Methods

n/a	Involved in the study	
☒	☐	ChIP-seq
☐	☒	Flow cytometry
☒	☐	MRI-based neuroimaging

Antibodies

Antibodies used

Anti-mouse CD45R , Clone: RA3-6B2, BioLegend, Cat#: 103224, RRID:AB_313007, dilution 1:400
Anti-mouse CD117, Clone: 2B8, BioLegend, Cat#: 105814, RRID:AB_313223, dilution 1:200
Anti-mouse CD11b, Clone: M1/70, BioLegend, Cat#: 101236, RRID:AB_11203704, dilution 1:200
Anti-mouse CD11b, Clone: M1/70, BD Bioscience, Cat#: 612800, RRID:AB_2870127, dilution 1:200
Anti-mouse CD11b, Clone: M1/70, BioLegend, Cat#: 101259, RRID: AB_2566568, dilution 1:200
Anti-mouse CD11b, Clone: M1/70, BioLegend, Cat#: 101243, RRID:AB_2561373, dilution 1:200
Anti-mouse CD11b, Clone: M1/70, BioLegend, Cat#: 101202, RRID:AB_312785, dilution 1:200
Anti-mouse CD11c, Clone: N418, BioLegend, Cat#: 117328, RRID:AB_2129641, dilution 1:200
Anti-mouse CD11c, Clone: N418, BioLegend, Cat#: 117333, RRID:AB_11204262, dilution 1:200
Anti-mouse CD11c, Clone: N418, BioLegend, Cat#: 117338, RRID: AB_2562016, dilution 1:200
Anti-mouse CD11c, Clone: N418, Biolegend, Cat#: 117302, RRID:AB_313771, dilution 1:200
Anti-mouse CD135, Clone: A2F10, BD Bioscience, Cat#: 562537, RRID: AB_2737639, dilution 1:200
Anti-mouse CD192, Clone: SA203G11, BioLegend, Cat#: 150603, RRID: AB_2566139, dilution 1:200
Anti-mouse CD192, Clone: 475301, BD Bioscience, Cat#: 750042, RRID: AB_2874259, dilution 1:200
Anti-mouse CD19, Clone: 6D5, BioLegend, Cat#: 115530, RRID:AB_830707, dilution 1:200
Anti-mouse CD150, Clone: 475301, BioLegend, Cat#: 115910, RRID: AB_493460, dilution 1:200
Anti-mouse CD3, Clone: 17A2, BioLegend, Cat#: 100222, RRID: AB_2242784, dilution 1:200
Anti-mouse CD131, Clone: JORO 50, BD Bioscience, Cat#: 559920, RRID: AB_397374, dilution 1:200
Anti-mouse CD45, Clone: I3/2.3 BioLegend, Cat#: 147710, RRID:AB_2563542, dilution 1:200
Anti-mouse CD45, Clone: 30-F11, BD Bioscience, Cat#: 564279, RRID: AB_2651134, dilution 1:200
Anti-mouse CD45, Clone: 30-F11, BD Bioscience, Cat#: 748370, RRID: AB_2872789, dilution 1:200
Anti-mouse CD45, Clone: 30-F11, BioLegend, Cat#: 103106, RRID: AB_312971, dilution 1:200
Anti-mouse CD45, Clone: 30-F11, eBioscience, Cat#: 14-0451-82, RRID:AB_467251, dilution 1:200
Anti-mouse CD45, Clone: 30-F11, BD Bioscience, Cat#: 553080, RRID:AB_394610, dilution 1:200
Anti-mouse CD45.1, Clone: A20, BD Bioscience, Cat#: 565212, RRID: AB_2722493, dilution 1:200
Anti-mouse CD45.2, Clone: 104, BD Bioscience, Cat#: 564880, RRID: AB_2738998, dilution 1:200
Anti-mouse CD16/32, Clone: 2.4G2, BDHorizon, Cat#: 741229, RRID: AB_2870783, dilution 1:100
Anti-mouse CD206, Clone: C068C2, BioLegend, Cat#: 141723, RRID: AB_2562445, dilution 1:200
Anti-mouse CD48, Clone: HM48-1, BioLegend, Cat#: 103439, RRID: AB_2650824, dilution 1:100
Anti-mouse CD90, Clone: 53-2.1, BioLegend, Cat#: 140325, RRID:AB_2650962, dilution 1:200
Anti-mouse CD64, Clone: X54-5/7.1, BioLegend, Cat#: 139314, RRID: AB_2563904, dilution 1:100
Anti-mouse CD64, Clone: X54-5/7.1, BioLegend, Cat#: 139302, RRID:AB_10613107, dilution 1:100
Anti-mouse CX3CR1, Clone: SA011F11, BioLegend, Cat#: 149035, RRID: AB_2629605, dilution 1:100
Anti-mouse F4/80, Clone: BM8, BioLegend, Cat#: 123141, RRID: AB_2563667, dilution 1:100
Anti-mouse IL-6, Clone: MP5-20F3, BD Biosciences, Cat#: 561367, RRID: AB_10679354, dilution 1:100
Anti-mouse Ly6C, Clone: HK1.4, BioLegend, Cat#: 128036, RRID:AB_2562353, dilution 1:200
Anti-mouse Ly6C, Clone: HK1.4, BioLegend, Cat#: 128037, RRID: AB_2562630, dilution 1:200
Anti-mouse Ly6C, Clone: HK1.4, BioLegend, Cat#: 128021, RRID: AB_10640820, dilution 1:200
Anti-mouse Ly6C, Clone: HK1.4, BioLegend, Cat#: 128016, RRID:AB_1732076, dilution 1:200
Anti-mouse Ly6C, Clone: HK1.4, BioLegend, Cat#: 128005, RRID:AB_1186134, dilution 1:200
Anti-mouse Ly6G, Clone: 1A8, BioLegend, Cat#: 127624, RRID:AB_10640819, dilution 1:200
Anti-mouse Ly6G, Clone: 1A8, BD Bioscience, Cat#: 747072, RRID: AB_2871828, dilution 1:200
Anti-mouse Ly6G, Clone: 1A8, BioLegend, Cat#: 127606, RRID: AB_1236494, dilution 1:200
Anti-mouse Ly6G, Clone: 1A8, BioLegend, Cat#: 127614, RRID: AB_2227348, dilution 1:200
Anti-mouse Ly6G, Clone: 1A8, BD Bioscience, Cat#: 741994, RRID: AB_2871294, dilution 1:200
Anti-mouse MERTK, Clone: 2B10C42, BioLegend, Cat#: 151510, RRID: AB_2832533, dilution 1:200
Anti-mouse MERTK, Clone: 108928, BD Bioscience, Cat#: 747890, RRID: AB_2872352, dilution 1:200
Anti-mouse MHC2, Clone: M5/114.15.2 , BioLegend, Cat#: 107635, RRID:AB_2561397, dilution 1:200
Anti-mouse MHC2, Clone: 2G9, BD Bioscience, Cat#: 750171, RRID: AB_2874376, dilution 1:200
Anti-mouse MHC2, Clone: M5/114.15.2, BioLegend, Cat#: 107622, RRID: AB_493727, dilution 1:200
Anti-mouse MHC2, Clone: M5/114.15.2 , BD Bioscience, Cat#: 750280, RRID: AB_2874471, dilution 1:200
Anti-mouse MHC2, Clone: M5/114.15.2, BioLegend, Cat#: 107602, RRID:AB_313317, dilution 1:200
Anti-mouse NK-1.1, Clone: PK136, BioLegend, Cat#: 108724, RRID:AB_830871, dilution 1:200
Anti-mouse Ly-6A/E, Clone: D7, Thermo Fisher Scientific, Cat#: 45-5981-82, RRID: AB_914372, dilution 1:200
Anti-mouse Siglec F, Clone: E50-2440, BD Biosciences, Cat#: 562757, RRID:AB_2687994, dilution 1:200
Anti-mouse Siglec F, Clone: E50-2440, BD Biosciences, Cat#: 740280, RRID: AB_2740019, dilution 1:200
Anti-mouse Siglec F, Clone: E50-2440, BD Biosciences, Cat#: 565934, RRID: AB_2739398, dilution 1:200
Anti-mouse Siglec F, Clone: E50-2440, BD Biosciences, Cat#: 565527, RRID: AB_2732831, dilution 1:200
Anti-mouse Siglec F, Clone: 1RNM44N, eBioscience, Cat#: 14-1702-82, RRID: AB_2572866, dilution 1:200
Anti-mouse TCR beta chain, Clone: H57-597, BioLegend, Cat#: 109219, RRID: AB_893626, dilution 1:400
Anti-mouse TER-119, Clone: TER-119, BioLegend, Cat#: 116223, RRID:AB_2137788, dilution 1:200
Anti-mouse CD16/32, Clone: 93, BioLegend, Cat#: 101320, RRID:AB_1574975, dilution 1:100
Anti-mouse CD16/32, Clone: 93, BioLegend, Cat#: 101325, RRID:AB_1953273, dilution 1:100
Anti-mouse CD34, Clone: SA376A4, BioLegend, Cat#: 152208, RRID: AB_2650766, dilution 1:100
Anti-mouse CD335, Clone: 29A1.4, BioLegend, Cat#: 137631, RRID: AB_2617040, dilution 1:200
Anti-mouse CD115, Clone: AFS98, BioLegend, Cat#: 135528, RRID: AB_2566523, dilution 1:100
Anti-mouse CD115, Clone: T38-320, BD Bioscience, Cat#: 749974, RRID: AB_2874201, dilution 1:100
Anti-mouse CD115, Clone: AFS98, BioLegend, Cat#: 135523, RRID: AB_2566459, dilution 1:100
Anti-mouse CD24, Clone: M1/69, eBioscience, Cat#: 12-0242-83, RRID: AB_465603, dilution 1:100
Anti-mouse CD24, Clone: M1/69, BD Bioscience, Cat#: 564664, RRID: AB_2716853, dilution 1:100
Anti-mouse CD24, Clone: M1/69, BioLegend, Cat#: 101822, RRID:AB_756048, dilution 1:100
Anti-mouse CD31, Clone: Mec13.3, BioLegend, Cat#: 102514, RRID:AB_2161031, dilution 1:200

	Anti-mouse CD326, Clone: G8.8, BioLegend, Cat#: 118218, RRID:AB_2098648, dilution 1:200<br>Anti-mouse Sca-1, Clone: D7, BioLegend, Cat#: 108120, RRID:AB_493273, dilution 1:100<br>Anti-mouse CD43, Clone: S7, BD Bioscience, Cat#: 741238, RRID: AB_2870790, dilution 1:200<br>Anti-mouse CD43, Clone: S7, BD Bioscience, Cat#: 560663, RRID: AB_1727479, dilution 1:200<br>Anti-mouse NKP46, Clone: 29A1.4, BioLegend, Cat# 137631, RRID:AB_2617040, dilution 1:200<br>Anti-mouse ApoE, Clone: EPR19392, Abcam, Cat#: ab183597, dilution: 1:50<br>Anti-mouse GPNMB, Clone: EPR18226-147, Abcam, Cat#: ab234529, dilution: 1:100<br>Anti-mouse CD326 (Ep-CAM), Clone: G8.8, BioLegend, Cat#: 118202, RRID:AB_1089027, dilution: 1:200<br>Anti-mouse Alpha Smooth Muscle Actin Antibody, Clone: 1A4, BioLegend, Cat#: 904601 RRID:AB_2565041, dilution: 1:200<br>Anti-mouse Sodium Potassium ATPase, Clone: EP1845Y, Abcam Cat#: ab76020, RRID:AB_1310695, dilution: 1:100<br>Anti-mouse CD140a (PDGFRa), Clone: APA5, eBioscience, Cat#: 14-1401-82, RRID:AB_467491, dilution: 1:200<br>Anti-mouse M-CSF, Clone: Polyclonal, ThermoFisher Scientific, Cat#: PA5-95279, RRID: AB_2807083, dilution: 1:50<br>Donkey Anti-Rat IgG (H+L) Antibody Cy3, Jackson ImmunoResearch, Cat#: 712-165-153, RRID:AB_2340667, dilution: 1:500<br>Donkey Anti-Goat IgG Antibody Cy3, Sigma-Aldrich, Cat#: AP180C, dilution: 1:500<br>Donkey anti-Rabbit IgG (H+L) Antibody Alexa Fluor 488, ThermoFisher Scientific, Cat#: A-21206, RRID: AB_2535792, dilution: 1:500<br>Donkey anti-Rabbit IgG (H+L) Antibody Alexa Fluor 568, ThermoFisher Scientific, Cat#: A10042, RRID: AB_2534017, dilution: 1:500<br>Donkey Anti-Rabbit IgG (H+L) Alexa Fluor 647, Abcam, Cat#: ab150075, dilution: 1:500<br>Donkey anti-Rat IgG (H+L) Antibody Alexa Fluor 488, ThermoFisher Scientific, Cat#: A-21208, RRID:AB_141709, dilution: 1:500<br>Ultra-LEAF™ Purified anti-mouse CD115 (CSF-1R) Antibody, BioLegend, Cat#: 135541, RRID:AB_2832485<br>Ultra-LEAF™ Purified Rat IgG2a κ Isotype Ctrl Antibody, BioLegend, Cat#: 400573<br>Anti-mouse α-SMA, clone: 1A4, Sigma-Aldrich, Cat#: F3777, RRID:AB_476977, dilution: 1:200<br>Anti-mouse EEA1, Clone: F.43.1, Thermo Fisher Scientific, Cat#: MA514794, RRID:AB_10985824, dilution: 1:100<br>Anti-mouse LAMP1, Clone: LY1C6, ThermoFisher Scientific, Cat#: MA1164, RRID:AB_2536869, dilution: 1:100<br>Anti-mouse Calreticulin, Clone: polyclonal, Themo Fisher Scientific, Cat#: PA3900, RRID:AB_325990, dilution: 1:200
Validation	All antibodies used in this study are commercially available and validated by the manufacturer. Validation data are available on the manufacturer's website. Clones were titrated in house on the tissue of interest and validated using single stain or FMO controls.

Animals and other research organisms

Policy information about studies involving animals; ARRIVE guidelines recommended for reporting animal research, and Sex and Gender in Research

Laboratory animals	All mouse strains used in this study have a C57BL/6 background and were bred in the animal facility of the LIMES Institute, University of Bonn, Germany or Center for Translational Cancer Research, Klinikum rechts der Isar, Technical University of Munich, Germany. Experiments were conducted using male mice aged 8-12 weeks. Mice were housed in IVC mice cages under conventional conditions (12 h/12 h light/dark cycle, 22°C, 55-70% humidity), with ad libitum access to food and water. Wildtype C57BL/6J mice are bred in house. Ai14 (JAX: 007914), CCR2ko (JAX: 004999), CD45.1 (JAX: 002014), Dectin-1ko (JAX: 012337) and LysM Cre (JAX: 004781) animals were obtained from The Jackson Laboratories. ApoE flox mice were kindly provided by Prof. J. Heeren, CARD9ko mice were kindly provided by Prof. J. Ruland, Ms4a3Cre mice were kindly provided by Prof. F. Ginhoux.
Wild animals	No wild animals were used in this study.
Reporting on sex	Male mice were housed in IVC mice cages under conventional conditions (12 h/12 h light/dark cycle, 22°C), with ad libitum access to food and water.
Field-collected samples	No field-collected samples were used in this study.
Ethics oversight	All experiments were approved by government of North Rhine-Westphalia (licenses 84-02.04.2017.A347 and 81-02.04.2020.A454).

Note that full information on the approval of the study protocol must also be provided in the manuscript.

Plants

Seed stocks	*Report on the source of all seed stocks or other plant material used. If applicable, state the seed stock centre and catalogue number. If plant specimens were collected from the field, describe the collection location, date and sampling procedures.*
Novel plant genotypes	*Describe the methods by which all novel plant genotypes were produced. This includes those generated by transgenic approaches, gene editing, chemical/radiation-based mutagenesis and hybridization. For transgenic lines, describe the transformation method, the number of independent lines analyzed and the generation upon which experiments were performed. For gene-edited lines, describe the editor used, the endogenous sequence targeted for editing, the targeting guide RNA sequence (if applicable) and how the editor was applied.*
Authentication	*Describe any authentication procedures for each seed stock used or novel genotype generated. Describe any experiments used to assess the effect of a mutation and, where applicable, how potential secondary effects (e.g. second site T-DNA insertions, mosiacism, off-target gene editing) were examined.*

Flow Cytometry

Plots

Confirm that:

☒ The axis labels state the marker and fluorochrome used (e.g. CD4-FITC).

☒ The axis scales are clearly visible. Include numbers along axes only for bottom left plot of group (a 'group' is an analysis of identical markers).

☒ All plots are contour plots with outliers or pseudocolor plots.

☒ A numerical value for number of cells or percentage (with statistics) is provided.

Methodology

Sample preparation	BALF samples were obtained by flushing the lung 3x with 1 ml 1x PBS with 10 mM EDTA. Peripheral blood was collected into 3 ml 1x PBS supplemented with 10 mM EDTA. For bone marrow, one tibia and femur were flushed with FACS buffer and filtered through a 70 µm strainer. Lungs were removed and one half was digested for 45 min at 37° C in HBSS supplemented with 10% FCS, 0.2 mg/ml collagenase IV and 0.05 mg/ml DNase I and then manually homogenized and filtered through a 70 µm strainer.<br>Pellets of single cell suspensions were resuspended in the antibody mix and incubated at 4°C. For the bone marrow, lung and blood, red blood cell lysis was perfomed. All samples were stained for life/death with DRAQ7 (1:1000 in FACS buffer) before acquisition.
Instrument	BD FACS Symphony A5, BD Aria III for sorting
Software	BD FACS Symphony: Diva software version 9.1, BD Aria III: Diva software 8.0.1
Cell population abundance	After cell sorting, the purity of the sorted populations was >90%
Gating strategy	For all analyzed tissues, cells were selected based on FSC-A and SSC-A. Doublets were excluded by FSC-A vs. FSC-H and SSC-A vs. SSC-H. CD45+ immune cells were selected by SSC-A vs. CD45, followed by exclusion of dead DRAQ7+ and lineage (B220, CD19, CD3ε, Nk1.1,Ter-119, TCR-beta, Nkp46 in APC-Cy7)+ cells. From CD45+ Lin- DRAQ7- cells, the respective populations were gated.<br>In the lung and BALF, AM were identified by CD64, MerTk and SiglecF expression. Gating strategy for further separation into CD11b- and CD11b+ AM is included in the supplementary information. According to this strategy, CD11b- and CD11b+ AM were sorted for the in vitro restimulation assay with LPS.<br>For single cell RNA sequencing, SSC high, Lin- (B220, CD19, CD3ε, Nk1.1, Ter-119), DRAQ7- singlets were sorted from the BALF.

☒ Tick this box to confirm that a figure exemplifying the gating strategy is provided in the Supplementary Information.
